# Slow radiological improvement and persistent low-grade inflammation after chemotherapy in tuberculosis patients with type 2 diabetes

**DOI:** 10.1186/s12879-020-05473-x

**Published:** 2020-12-07

**Authors:** Akhirunnesa Mily, Protim Sarker, Inin Taznin, Delwar Hossain, Md. Ahsanul Haq, S. M. Mostofa Kamal, Birgitta Agerberth, Susanna Brighenti, Rubhana Raqib

**Affiliations:** 1 Center for Infectious Medicine (CIM), Department of Medicine Huddinge, ANA Futura, Karolinska Institutet, Stockholm, Sweden; 2 Infectious Diseases Division, International Centre for Diarrhoeal Disease Research, Dhaka, Bangladesh; 3 Respiratory Medicine, Bangladesh Institute of Research and Rehabilitation in Diabetes, Endocrine and Metabolic Disorders, Dhaka, Bangladesh; 4 National Institute of the Diseases of the Chest and Hospital, Dhaka, Bangladesh; 5 Clinical Microbiology, Department of Laboratory Medicine (Labmed), ANA Futura, Karolinska Institutet, Stockholm, Sweden

**Keywords:** Anti-inflammatory cytokine, Diabetes mellitus, IL-10, Pulmonary pathology, Sputum culture, TB score

## Abstract

**Background:**

Diabetes mellitus type 2 (DM) may impede immune responses in tuberculosis (TB) and thus contribute to enhanced disease severity. In this study, we aimed to evaluate DM-mediated alterations in clinical, radiological and immunological outcomes in TB disease.

**Methods:**

Newly diagnosed pulmonary TB patients with or without DM (TB *n* = 40; TB-DM n = 40) were recruited in Dhaka, Bangladesh. Clinical symptoms, sputum smear and culture conversion as well as chest radiography were assessed. Peripheral blood and sputum samples were collected at the time of diagnosis (baseline) and after 1, 2 and 6 months of standard anti-TB treatment. Blood samples were also obtained from healthy controls (*n* = 20). mRNA expression of inflammatory markers in blood and sputum samples were quantified using real-time PCR.

**Results:**

The majority of TB-DM patients had poor glycemic control (HbA1c > 8%) and displayed elevated pulmonary pathology (*P* = 0.039) particularly in the middle (*P* < 0.004) and lower lung zones (*P* < 0.02) throughout the treatment period. However, reduction of clinical symptoms and time to sputum smear and culture conversion did not differ between the groups. Transcripts levels of the pro-inflammatory cytokines IL-1β (*P* = 0.003 at month-1 and *P* = 0.045 at month-2) and TNF-α (*P* = 0.005 at month-1) and the anti-inflammatory cytokine IL-10 (*P* = 0.005 at month-2) were higher in peripheral blood after anti-TB treatment in TB-DM compared to TB patients. Conversely in sputum, TB-DM patients had reduced CD4 (*P* < 0.009 at month-1) and IL-10 (*P* = 0.005 at month-1 and *P* = 0.006 at month-2) transcripts, whereas CD8 was elevated (*P* = 0.016 at month-2). At 1- and 2-month post-treatment, sputum IL-10 transcripts were inversely correlated with fasting blood glucose and HbA1c levels in all patients.

**Conclusion:**

Insufficient up-regulation of IL-10 in the lung may fuel persistent local inflammation thereby promoting lung pathology in TB-DM patients with poorly controlled DM.

## Background

The convergence of tuberculosis (TB) and type 2 diabetes mellitus (DM) has emerged as a serious threat to global TB control. Type 2 DM patients are estimated to have a 3-fold higher risk of developing active TB infection compared to non-diabetic individuals [1]. Moreover, it is predicted that by 2030, around 80% of all type 2 DM patients will be living in developing countries, where there is also a high incidence of TB [2]. In the WHO Global report 2019, Bangladesh is ranked seventh in the list of countries with a high-burden for TB, accounting for 4% of global cases. Concurrently, DM is dramatically increasing in Bangladesh and according to the International Diabetes Federation the prevalence was around 7% amongst adults in 2017. TB is a chronic infection caused by *Mycobacterium tuberculosis* (Mtb), while DM is a non-communicable metabolic disease. Poor glycemic control is considered a major risk factor for the development of TB in DM patients [3] and may contribute to alterations in peripheral, as well as local, immune cell responses at the site of Mtb infection.

Several studies have demonstrated that at the end of 2 months intensive phase anti-TB treatment diabetic TB patients remain sputum positive more often than TB patients without DM [4–8]. However, other studies failed to detect differences between TB-DM and TB patients in sputum smear or culture conversion [9, 10]. TB-DM co-morbidity at the time of TB diagnosis has frequently been associated with a hyper-inflammatory cytokine profile [11, 12], and recently a plasma biomarker assay demonstrated that this systemic inflammatory state persisted in TB-DM patients throughout effective anti-TB treatment [13]. Excess systemic circulation of pro-inflammatory cytokines including IL-8, IL-12p70, and TNF-α and a reduction in anti-inflammatory cytokines such as IL-10, could promote non-resolving inflammation in TB-DM patients with unfavorable effects on TB disease progression and anti-TB treatment outcomes [13]. While TNF-α and IL-1β stimulate both antimicrobial and other protective effects in immune cells, prolonged and/or excess expression of these cytokines can result in overt pathology and tissue damage [14, 15]. IL-10 counteracts these effects by inhibiting the generation of pro-inflammatory mediators in immune cells [16]. However, Mtb may also exploit the regulatory function of IL-10 to suppress cellular immune responses [17, 18]. As such, it has been suggested that persistently higher bacillary burden and more severe TB disease in DM patients is attributed to a delay in the adaptive immune response to Mtb in the lung or in the lung-draining lymph nodes [19, 20]. Impaired T helper 1 (Th1) responses and a lower Th1-to-Th2 cytokine ratio in peripheral blood could result in a Th2-biased immune response in TB-DM patients [21–23]. Moreover, it has been shown that the cytotoxic activity of CD8+ T cells is altered in patients with TB-DM comorbidity [24, 25]. Despite these findings, it has also been reported that several of these immunological changes may occur without loss of antimicrobial activity or reduction of bacterial loads [26–28]. Hence, there is still no scientific consensus regarding the role of DM in the progression of TB disease.

In this study, we aimed to evaluate the effects of DM on TB-associated local and systemic inflammation, as well as clinical and radiological manifestations of TB disease at baseline and after the start of standard anti-TB treatment.

## Methods

### Patients and treatment

This observational study was conducted in two tertiary care hospitals in Dhaka from June 2014 to May 2017. TB patients (*n* = 40) were recruited from the National Institute of the Diseases of the Chest and Hospital (NIDCH) and TB-DM patients (n = 40) from the Bangladesh Institute of Research and Rehabilitation for Diabetes, Endocrine and Metabolic Disorders (BIRDEM). Age- and sex-matched healthy individuals (*n* = 20) were randomly recruited as controls. Diabetes status was defined (according to WHO criteria) as patients with either a fasting blood glucose concentration ≥ 7 mmol/liter or HbA1c ≥ 6.5%. Inclusion criteria: adult males and females, age 18–60 years, newly diagnosed TB using sputum-smear microscopy and/or GeneXpert MTB/RIF and a history of diabetes for ≤5 years (TB-DM group). Exclusion criteria: previous history of TB, systemic or miliary TB, > 1 week of anti-TB treatment, pregnancy, history of type 2 DM for > 5 years, and concomitant illnesses such as cardiovascular, liver or kidney diseases, cancer or HIV infection. It was decided to exclude patients with DM disease > 5 years in order to avoid the multiple accompanying vascular complications that are associated with long-term DM such as cardiovascular disease, retinopathy, neuropathy and nephropathy [29].

Standard anti-TB treatment involved directly observed therapy short-course (DOTS) regimen that consists of isoniazid, rifampicin, pyrazinamide and ethambutol for 2 months followed by isoniazid and rifampicin for a subsequent 4 months. At enrollment, DM patients received different types of anti-diabetic medications including metformin hydrochloride (*n* = 3), insulin (*n* = 29), a combination of metformin and insulin (*n* = 7), or other drugs (*n* = 1).

### Clinical samples and procedures

After enrolment, socio-economic status (SES), body weight, height, clinical history including duration of illness, history of contact with active TB cases, *Bacillus Calmette–Guérin* (BCG) vaccination, smoking habits and medication history (TB-DM patients) were recorded. SES was estimated utilizing a wealth index generated through principal component analysis of household assets [30]. At baseline, and months-1, − 2 and − 6 after the initiation of standard anti-TB treatment, clinical evaluation (mentioned in Table 1 and Table S1) and radiological examinations were performed.

**Table 1:** Baseline characteristic of the study participants^a^

Variables	TB (*n* = 40)	TB-DM (*n* = 40)	*p*-value^b^	Healty Controls (*n* = 20)	*p*-value^c^	*p*-value^d^
Sex, Male (n, %)	29 (75.5)	39 (97.5)	0.0024	14 (70.0)	0.466	< 0.001
Age (years)	26.6 ± 7.6	40.1 ± 8.8	< 0.0001	32.5 ± 6.3	0.036	0.030
BMI (Kg/m2)	17.6 ± 2.7	21.7 ± 2.5	< 0.001	26.1 ± 3.9	< 0.001	< 0.005
Family SES						
1st tertile (poor), n (%)	19 (47.5)	8 (20.00)		0		
2nd tertile (middle), n (%)	13 (32.5)	15 (37.5)		3		
3rd tertile (rich), n (%)	8 (20.00)	17 (42.5)		17		
BCG vaccination status, n (%)	23 (57.5)	35 (87.5)	< 0.0025	18 (90.0)	0.023	0.775
History of contact with active cases	19 (54.3%)	16 (45.7%)	0.255	–		
Duration of Symptom (days)	61 (30, 105.5)	75.5 (60, 121)	0.087	–		
Sputum smear results (AFB), n (%)						
^e^AFB negative	0	2 (5%)		–		
1+ AFB	9 (22.5%)	10 (25.0%)		–		
2+ AFB	11 (27.5%)	9 (22.5%)		–		
3+ AFB	20 (50.0%)	19 (47.5%)		–		

^a^Quantitative data are presented as median ± IQR; Categorical data are presented as n (%). Statistical analysis comparing ^b^TB vs TB-DM, ^c^TB vs HC, and ^d^TB-DM vs HC was done using Chi-square, Kruskal-Wallis and Dunn’s post-test or the Mann-Whitney U-test ^e^Sputum samples positive in the GeneXpert MTB/RIF test. AFB: Acid-Fast Bacilli; BCG: *Bacillus Calmette–Guérin;* BMI: body mass index; SES: socioeconomic status

Fasting blood and sputum samples were collected from the patients at baseline and during follow up visits to the hospitals and specimens were brought to the Laboratory at the International Centre for Diarrhoeal Disease Research, Bangladesh (icddr,b). Healthy controls provided a fasting blood sample on one occasion. Whole blood was routinely analyzed for erythrocyte sedimentation rate (ESR) using an ESR analyzer (SRS 100/II, Greiner Bio-One GmbH, Kremsmunster, Austria) and complete blood count (CBC) using an automated hematology analyzer (XN-1000, Sysmex Corporation, Kobe, Japan). CBC assessment included total and differential blood cell counts (Table S2) and hemoglobin concentration. Peripheral blood mononuclear cells (PBMCs) and plasma were isolated from heparinized blood using Ficoll-Paque PLUS (GE Healthcare, Uppsala, Sweden) density gradient centrifugation. Blood glucose and HbA1c level were measured using a Clinical Chemistry Analyzer (Cobas C311, Roch Diagnostics GmbH, Mannheim, Germany). Plasma levels of the metabolic hormones insulin and C-peptide were measured by Luminex assay using a Bio-Plex Diabetes kit (Bio-Rad Laboratories, Inc. USA). The homeostatic model assessment of insulin resistance (HOMA-IR) was calculated based on the following formula: HOMA-IR = (fasting insulin [pmol/L] × fasting plasma glucose [mmol/L]) / 135 [31]. C-peptide reactivity-insulin resistance (CPR-IR) was calculated using the following formula: CPR-IR = 20 / (fasting C-peptide [nmol/L] x fasting plasma glucose [mmol/L]) [32]. Expectorated sputum samples were used for sputum-smear microscopy and culture. PBMCs and an aliquot of sputum were stored at − 80 °C in 1 ml of RNA*later* (Qiagen GmbH, Hilden, Germany) for mRNA analysis.

### Clinical composite TBscore

The Bandim TBscore [33] was applied for assessment of the following signs and symptoms of TB; cough, hemoptysis, dyspnoea, chest pain, night sweats, anemia, fever, body mass index (BMI) and mid upper arm circumference (MUAC). These signs of TB and typical symptoms were recorded and scored as present (1) or absent (0) [34]. In contrast to the Bandim TBscore, data on tachycardia and lung auscultation were not included and consequently this TBscore had a maximum value of 11 points.

### Mtb sputum-smear microscopy, sputum culture and the Xpert MTB/RIF assay

Sputum-smear microscopy was conducted at the NIDCH or BIRDEM, while Mtb culture was performed in the Mycobacteriology laboratory at icddr,b. Ziehl-Neelsen staining was performed to detect and grade acid-fast bacilli by smear microscopy. Decontaminated sputum pellets were cultured on Lowenstein-Jensen (L-J) slants and were examined weekly until Mtb colonies were detected to confirm the TB diagnosis. The Xpert MTB/RIF assay was performed using sputum collected from all TB patients at NIDCH or BIRDEM to rule out Mtb resistance to rifampicin according to the national TB program (NTP) guidelines in Bangladesh. Drug susceptibility testing was not performed in the isolated Mtb strains as this is not recommended by the NTP.

### Chest X-ray analysis

For lung pathology analysis, a semi-quantitative visual scoring system of 2-dimensional chest X-rays was used as previously reported [35, 36], although with some modifications. For each patient, the two lungs were divided into three zones: upper, middle and lower. Presence of nodules, patchy or confluent consolidations, and areas of cavitation were recorded in each of the three zones. The effusion volume, extent of opacification, cavitation or additional pathology were graded as the percentage (%) of the affected lung. Each of the three zones in the two lungs could be graded as having a maximal pathological involvement of 100%, and therefore the total % lung involvement in the upper, middle or lower zones, respectively, could be a maximum of 100 + 100 = 200%. Accordingly, in each patient, the total % lung involvement comprising all three zones in both lungs could be a maximum of 3 × 100 × 2 = 600%.

### mRNA extraction and real-time PCR

mRNA analysis of PBMCs and sputum cells was conducted to assess inflammation in the peripheral circulation compared to the local site of Mtb infection. This analysis was performed on a sub-set of the study patients from whom longitudinal and matched blood and sputum samples could be collected. This selection was random and representative of median HbA1c levels and chest X-ray scores in the total patient cohort. mRNA was extracted using Ambion RiboPure RNA extraction kit (Life Technologies, Vilnius, Lithuania). cDNA conversion was performed using a SuperScript cDNA synthesis kit (Invitrogen, Carlsbad, CA). The target genes (CD4, CD8, IL-10, IL-1β, TNF-α and matrix metalloproteinase (MMP)-9), and the house-keeping gene (18srRNA) were amplified using Taqman gene expression master mix and primers in the QuantStudio 5 Real-Time PCR System (Applied Biosystems, Foster City, CA). The cycle threshold (Ct) values for the target genes were normalized to 18srRNA and relative mRNA expression was determined using the 2^−ΔΔCT^ method. mRNA analysis was performed using sputum samples collected at baseline, month-1 and month-2, as by 6 months, most TB patients were unable to produce sputum.

### Sample size calculation

This was an exploratory pilot study where our aim was to investigate immunological responses in patients with TB-DM compared to patients with TB alone. In general, simulation studies have identified a minimum sample size requirement of > 20 for conducting powerful parametric analyses, even when the data is non-normally distributed. However, human clinical data tends to be variable when involving less than 30 patients or volunteers in their respective groups. Based on our previous experience with TB studies in high-endemic countries [37–41], inclusion of > 20 patients in each sub-group enabled parametric analyses, while smaller sub-group analyses were performed using non-parametric methods. Assuming an initial sample size of 30 and accounting for a loss to follow-up of 15% and low quality or insufficient amount of clinical material in another 10% of the patients, we used a sample size of *n* = 40 in the TB-DM and TB groups.

### Statistical analysis

Data is presented as mean and standard error of mean (SEM) or median and interquartile range (IQR) for continuous data, and numbers with percentages for categorical variables. Statistical significance was determined using non-parametric Kruskal-Wallis test with Dunn’s post-test (comparing more than two unmatched groups), Mann-Whitney test (comparing two unmatched groups) and Friedman test with Dunn’s multiple comparison test (comparing non-parametric one-way repeated measurements). The Chi-square test was used to compare the proportion of patients with TB symptoms between the TB and TB-DM groups at baseline. Multivariable regression model and GEE (related to lung pathology only) analysis were used to estimate the difference in outcome variables between the TB-DM and TB groups, and data was adjusted for covariates (age, sex, BCG vaccination status, baseline BMI and SES score). The GEE model (beta (β) value and 95% confidence interval (CI)) were created with an interaction (follow-up month × groups) to analyze changes in blood hemogram markers and percent lung involvement across time by group status. This analysis was performed at baseline, and during three follow up visits with the following predictors: Time (months 0, 1, 2 and 6), group, and an interaction term of these two (visit month × group). Covariates that influenced the model R^2^ by 5% or more were selected to avoid collinearity. To evaluate changes within groups in the TBscore, BMI, radiological feature, blood glucose, HbA1c and CBC, analyses were performed using 2-way repeated measure Analysis of variance (ANOVA). Spearman’s correlation test was used for the correlation analyses. Stata/IC (v.13, Stata Corp., LP, College Station, Texas, USA) and GraphPad Prism 8.3.0 were used for statistical analysis. A *P*-value < 0.05 was considered as significant.

## Results

### Baseline characteristics

**Fig. 1:**
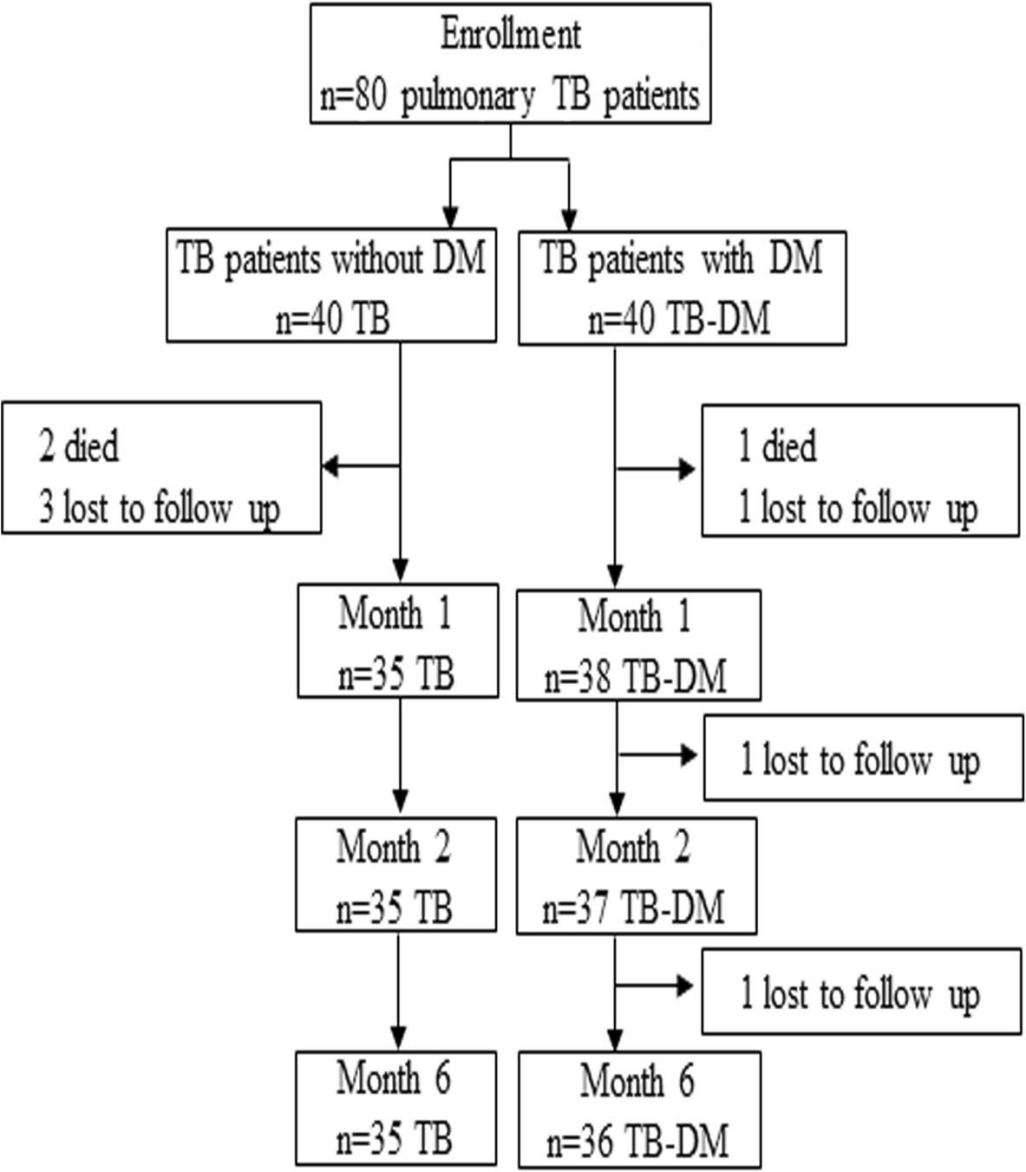
Flow chart illustrating enrollment and follow-up of TB and TB-DM patients

The enrollment of TB patients with or without DM is illustrated in Fig. 1 and the demographic, clinical and hematological profiles are shown in Table 1 and in Tables S1 and S2. Forty patients were enrolled in each group with follow-up of 35 TB and 36 TB-DM patients up to 6 months of anti-TB treatment (Fig. 1). None of the patients’ Mtb isolates were resistant to rifampicin as determined by GeneXpert MTB/RIF analysis and all patients received standard anti-TB therapy according to the NTP guidelines. TB-DM patients were significantly older with better SES compared to TB patients. BMI was significantly higher in the TB-DM cohort compared to the TB group, although both groups had a lower mean BMI compared to the healthy controls. Blood hemogram at enrolment demonstrated that ESR, total white blood cell (WBC) and neutrophil counts were significantly elevated, whereas lymphocyte counts were significantly reduced in both TB and TB-DM patients compared to healthy controls (Table S2). Differences seen in the TB patients compared to TB-DM patients included significantly higher hemoglobin (Hb) in the TB-DM group, whereas the monocyte count was higher in the TB group.

### Poor glycemic control in TB-DM patients

**Fig. 2:**
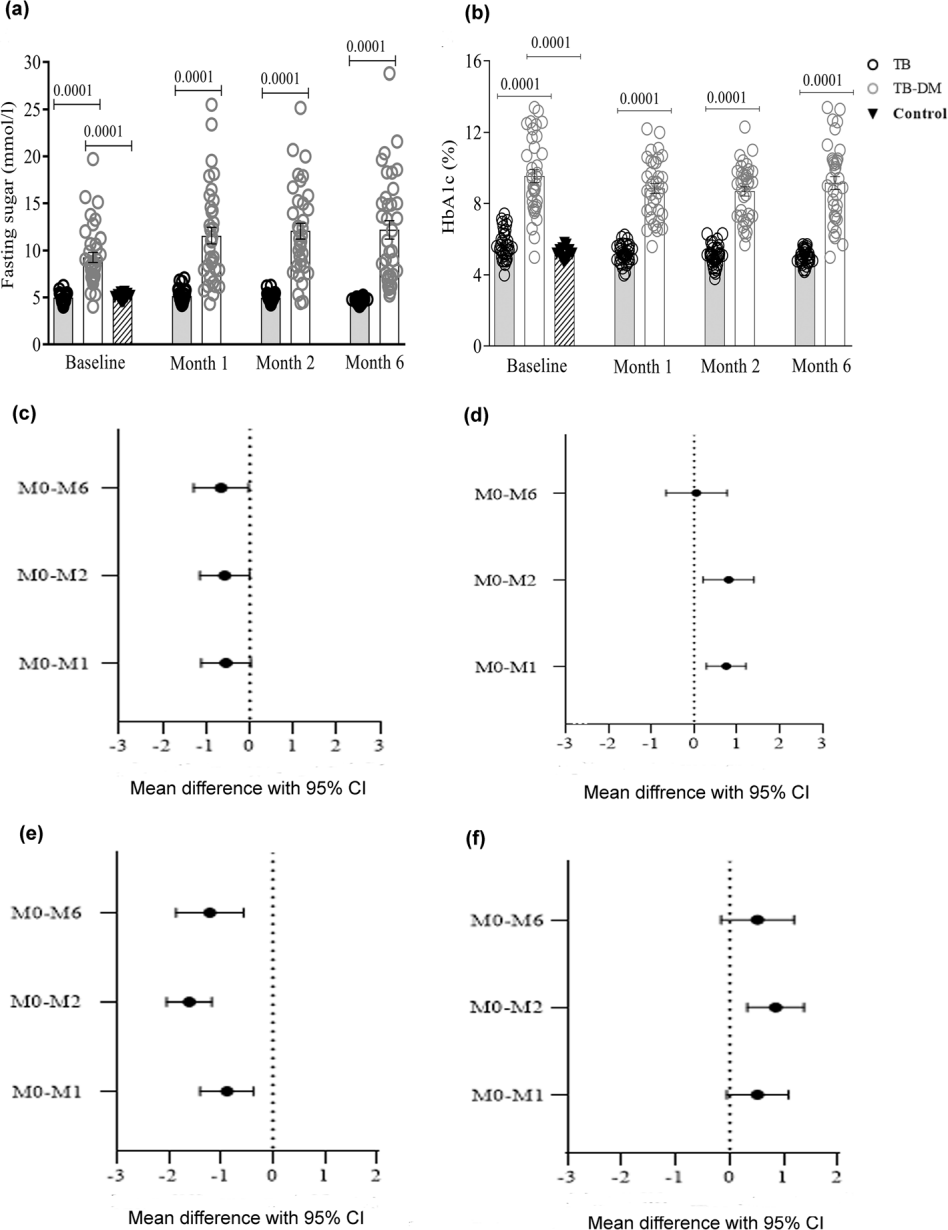
Glycemic markers in TB and TB-DM patients and in healthy controls (**a**, **b**), and correlation of glycemic markers (**c**, **d**), homeostatic model assessment of insulin resistance (HOMA-IR) (**e**) and C-peptide reactivity-insulin resistance (CPR-IR) (**f**) with time-since–treatment in TB-DM patients. Fasting blood glucose levels (mmol/L) and HbA1c concentrations (%) were assessed in TB (*n* = 35) and TB-DM (*n* = 36) patients at enrolment and after 1, 2 and 6 months of anti-TB treatment, and once in healthy controls (*n* = 20). Plasma insulin levels (pg/mL) and plasma C-peptide levels (pg/mL) were assessed in TB-DM (*n* = 35) patients at enrolment and after 1, 2 and 6 months of anti-TB treatment. HOMA-IR was calculated using the formula: HOMA-IR = (fasting insulin [pmol/L] × fasting plasma glucose [mmol/L]) / 135). CPR-IR was calculated using the formula: CPR-IR = 20 / (fasting C-peptide [nmol/L] x fasting plasma glucose [mmol/L]). Data are presented as mean withstandard error of mean (**a**, **b**) or as mean difference with 95% confidence interval (**c**, **d**, **e**, **f**). Statistical differences were calculated using multivariate regression, adjusting for age, sex, SES score, BCG vaccination status and baseline BMI (**a**, **b**), and Friedman's test (**c**, **d**, **e**, **f**). *p < 0.05 was considered significant. BMI: body mass index; BCG: *Bacillus Calmette–Guérin*; HbA1c: glycosylated hemoglobin; SES: socio economic status

TB-DM patients had significantly higher levels of fasting blood glucose and HbA1c at enrollment compared to TB patients and healthy controls (Fig. 2a-b). Blood glucose levels were further elevated in TB-DM patients after the initiation of anti-TB chemotherapy compared to those recorded at baseline (Fig. 2a). Likewise, HbA1c remained elevated in TB-DM patients at the follow up visits (Fig. 2b). However, neither fasting glucose nor HbA1c levels correlated with time-since-treatment (Fig. 2c-d). Insulin resistance calculated using insulin levels (HOMA-IR) increased in the TB-DM patients after the start of anti-TB treatment (Fig. 2e). C-peptide based insulin resistance (CPR-IR) in the TB-DM group was significantly reduced at month 2 but increased again by month 6 (Fig. 2f). These results suggested that TB-DM patients maintained poor glycemic control at TB diagnosis and throughout anti-TB treatment.

### Similar changes in signs and symptoms of TB, and in sputum culture conversion in TB and TB-DM patients after anti-TB treatment

**Fig. 3:**
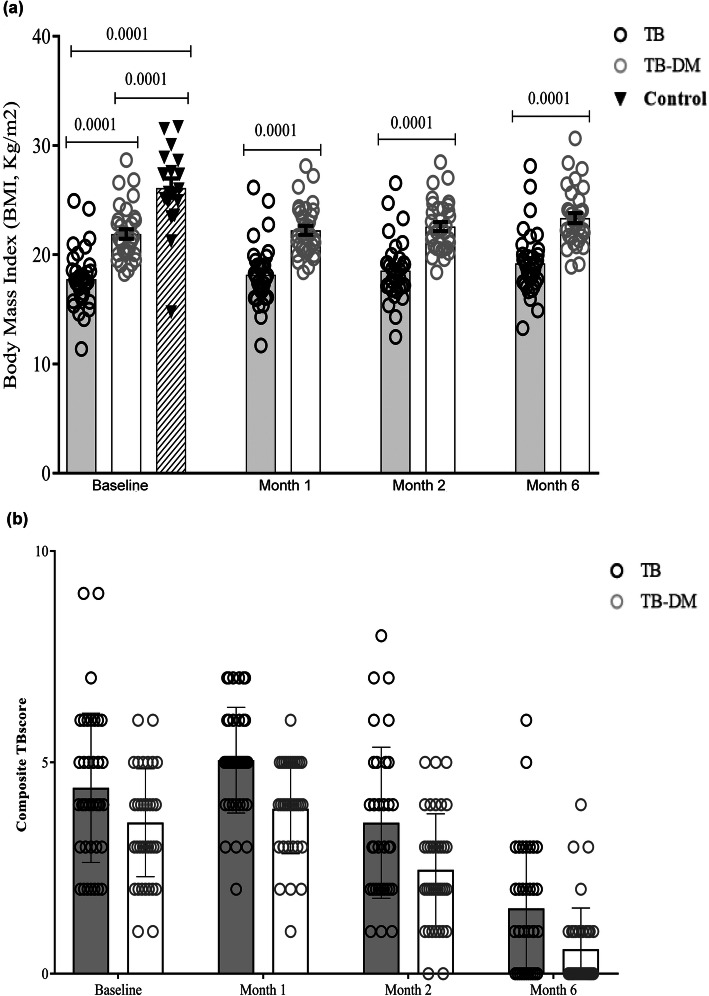
BMI and composite TBscore in TB and TB-DM patients. **a** BMI (kg/m2) and **b** composite TBscore were assessed in TB *(n* = 35) and TB-DM (*n* = 36) patients at enrolment and after 1, 2 and 6 months of anti-TB treatment. BMI was also assessed on one occasion in healthy controls (*n* = 20). Data is presented as mean with standard error of mean. Statistical differences were calculated using adjusted multivariate regression. The regression model was adjusted for age, sex, SES score and BCG vaccination status. *p < 0.05 was considered significant. BMI: body mass index; BCG: *Bacillus Calmette–Guérin*; SES: socio economic status

All patients experienced typical TB symptoms such as fever and a cough (Table S1). Symptoms such as chest pain, loss of appetite, nausea, anemia and low BMI were more common in TB patients, while night sweats were experienced more often by TB-DM patients. BMI steadily increased in both patient groups after anti-TB treatment but remained significantly higher in TB-DM patients compared to the TB patients (Fig. 3a). Both patient groups had a significant reduction in the composite TBscore after the start of anti-TB chemotherapy and there was no significant difference between patient groups at any time point (Fig. 3b).

The majority of patients with TB (in both groups) had positive Mtb sputum smear (Table 1) and culture results. After 1 month of anti-TB treatment, the proportion of TB-DM patients with negative Mtb culture results was similar to that seen in the TB patients (26/36 = 72% versus 20/35 = 57%, *P* = 0.11 by chi-square test). By 2 months, 97 and 95% of TB-DM and TB patients, respectively had a negative Mtb sputum culture (*P* = 0.98), while all patients were Mtb culture negative after 6 months of treatment. Treatment outcome was similar in both patient groups; 90% of the TB-DM patients and 87.5% of the TB patients were cured of TB after 6 months of anti-TB treatment.

### Slower radiological improvement in TB-DM patients after anti-TB treatment

**Fig. 4:**
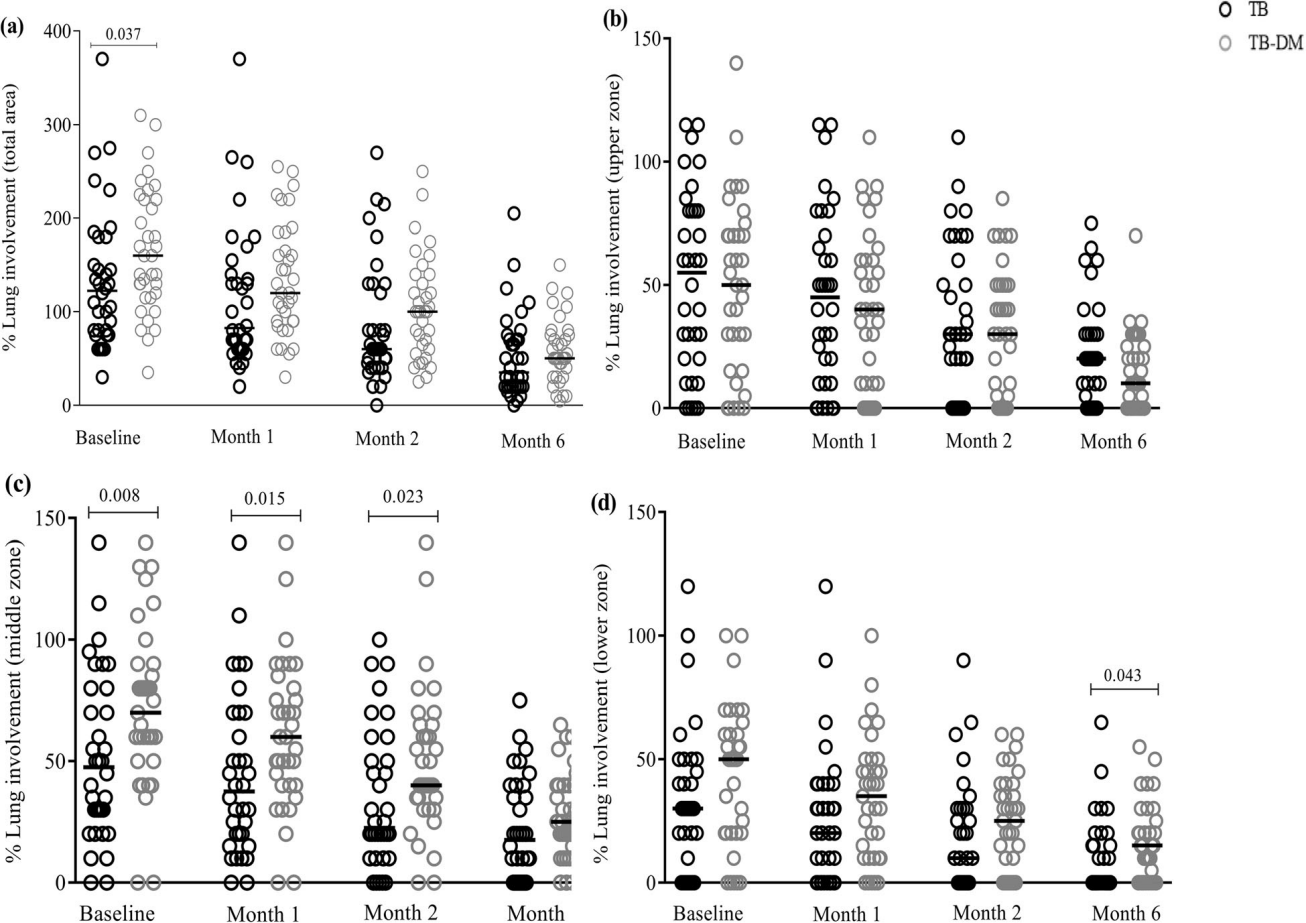
Chest X-ray findings in TB and TB-DM patients. **a** total percent lung involvement (combined pathology in upper, middle and lower zones of the left and right lungs), **b** percent lung involvement in the upper zone, **c** percent lung involvement in the middle zone, and **d** percent lung involvement in the lower zone was assessed in TB (*n* = 35) and TB-DM (*n* = 36) patients at enrolment and after 1, 2 and 6 months of anti-TB treatment. Note that each of the three zones in the two lungs could have a maximum of 100% pathological involvement, and therefore the total percent lung involvement in the upper, middle or lower zones, respectively, could be maximum 100 + 100 = 200% (**b**-**d**), while the total percent lung involvement including all three zones in both lungs in a patient could be maximum 3 × 100 × 2 = 600% (**a**). Data is presented as median values. Statistical differences between the TB and TB-DM group were calculated using the Mann-Whitney U-tests, and *p* < 0.05* was considered significant

Assessment of pulmonary pathology using chest X-ray and quantification of inflammatory lesions in the different zones of the left and right lungs revealed that anti-TB treatment gradually reduced pulmonary inflammation in both TB and TB-DM patients (Fig. 4). At baseline, total lung involvement was significantly higher in TB-DM compared to TB patients, but this difference was absent at follow up as assessed by Mann-Whitney U-tests (Fig. 4a). No differences in lung involvement in the upper zone were detected when comparing the two groups (Fig. 4b). However, middle zone lung involvement was significantly higher in the TB-DM patients at baseline and at months-1 and -2 compared to the TB group (Fig. 4c) and the same difference was seen in the lower zones at month-6 (Fig. 4d). Multivariate regression analysis showed significantly higher levels of percent lung involvement in the whole lung, as well as in the middle and lower zones in TB-DM patients compared to TB patients at baseline (Table S3). Longitudinal analysis using the GEE model also showed that when compared to TB patients, TB-DM patients had significantly elevated pulmonary involvement in the middle and lower zones and in the whole lung (Table S3).

### Lung pathology is associated with hemogram parameters and HbA1c levels

After anti-TB treatment, both patient groups showed a decrease in ESR, WBC, neutrophil, monocytes and platelet counts, NLR, monocyte-to lymphocyte ratios (MLR) and also PLR, however there were increased hemoglobin levels and lymphocyte counts (Table S2). Multiple regression analysis showed that the TB-DM group had a significantly higher WBC count at month-6 compared to TB patients (Table S2).

Lung pathology in TB-DM patients was inversely associated with hemoglobin levels and lymphocyte counts, and positively associated with ESR, WBC, neutrophil count, platelet count and PLR (Table 2). In TB patients, there was an inverse association between pathological lung involvement and hemoglobin levels and a positive association with WBC count, platelet count and PLR (Table 2). When both patient groups were combined, a strong association was seen between HbA1c and percent lung involvement (β = 1.01, 95% CI = -0.19, 2.22; *P* = 0.09).

**Table 2:** Longitudinal association of lung involvement with TBscore and blood hemogram markers in TB and TB-DM patients^a^

	% lung involvement^b^			
	TB (*n* = 35)		TB-DM (*n* = 36)	
	β-coefficient (95% CI)	*p*-value^c^	β-coefficient (95% CI)	*p*-value^c^
TBscore	−0.003(−0.03, 0.02)	0.802	0.02(−0.01, 0.04)	0.143
ESR	0.03(− 0.0003, 0.05)	0.053	0.06 (0.03, 0.09)	< 0.001
Hb	−0.34(− 0.66, − 0.01)	0.041	−1.15(−1.80, − 0.49)	0.001
WBC	0.32 (0.08, 0.56)	0.008	0.41 (0.13, 0.69)	0.004
Lymphocyte	−0.05(− 01.2, 0.02)	0.173	− 0.14(− 0.23, − 0.05)	0.002
Neutrophils	0.06(− 0.003, 0.13)	0.060	0.11 (0.04, 0.18)	0.002
Monocyte	−0.22(− 0.64, 0.20)	0.298	0.01(− 0.40, 0.41)	0.980
Platelet	0.02 (0.01, 0.04)	0.001	0.02 (0.01, 0.03)	0.005
NLR	0.09(−0.02, 0.21)	0.121	0.10(−0.11, 0.31)	0.353
MLR	8.53(−2.43, 19.49)	0.127	6.07(−0.78, 12.93)	0.082
PLR	0.17 (0.07, 0.27)	0.001	0.12 (0.04, 0.20)	0.003

^a^Data were analyzed using Generalized estimating equation (GEE), and the results are expressed as beta β-coefficient and 95% confidence interval (CI). The GEE model was adjusted by age, sex, baseline BMI, SES score, BCG vaccination status and time (to reduce multicollinearity)

^b^Each of the three zones in the two lungs could have a maximum of 100% pathological involvement, and therefore the total % lung involvement in the upper, middle or lower zones respectively could be maximum 100 + 100 = 200%, while the total % lung involvement including all three zones in both lungs in a patient could be maximum 3 × 100 × 2 = 600%

^c^p-values demonstrate the significant association of lung involvement and the listed variables in both TB and TB-DM patients

*BCG Bacillus Calmette–Guérin, BMI* body mass index, *CI* confidence interval, *ESR* erythrocyte sedimentation rate, *Hb* hemoglobin, *MLR* monocyte-to-lymphocyte ratio, *NLR* neutrophil-to-lymphocyte ratio, *PLR* platelet-to-lymphocyte ratio, *SES* socioeconomic status, *WBC* white blood cell

### mRNA expression profiling suggests higher levels of inflammation in TB-DM patients after anti-TB treatment

**Fig. 5:**
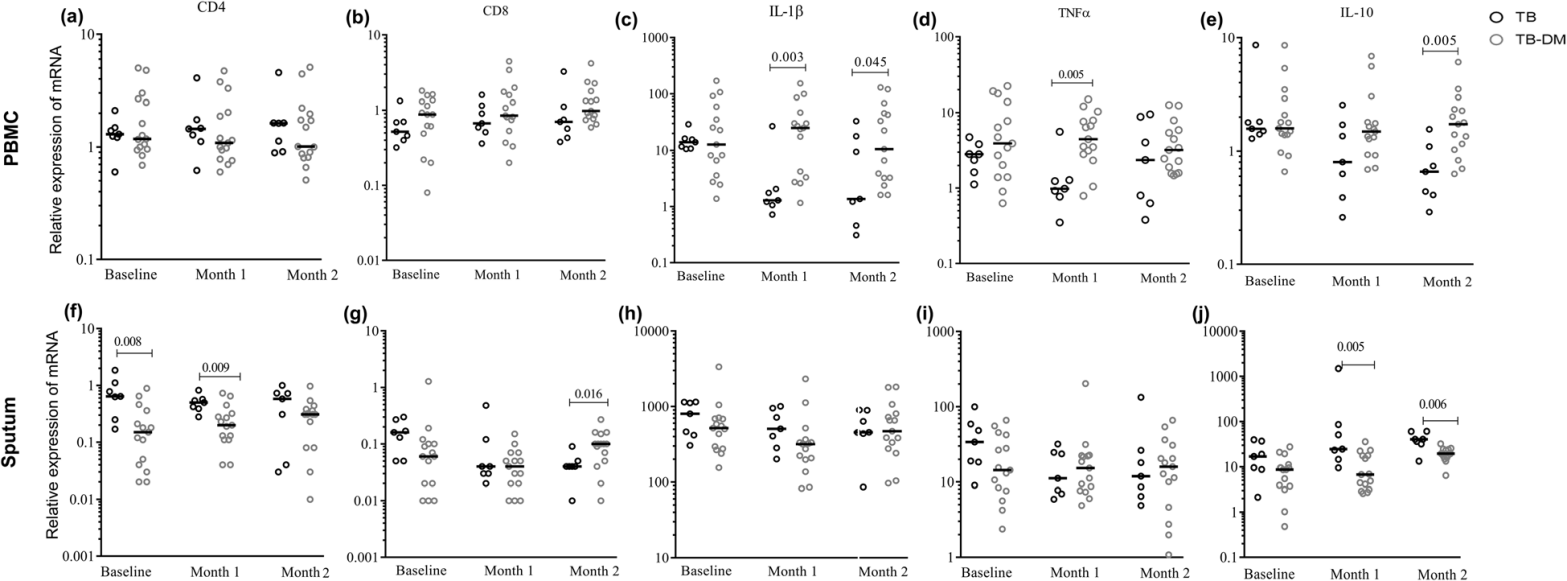
mRNA profiling of PBMCs and sputum cell samples from TB and TB-DM patients. Quantitative mRNA expression of CD4, CD8, IL-1β, TNF-α and IL-10 in PBMCs (**a**-**e**) and sputum cell samples (**f-j**) from TB (*n* = 7) and TB-DM (*n* = 15) patients were analyzed at enrolment and after 1 and 2 months of anti-TB treatment. Data is presented as median values and statistical differences between the TB and TB-DM group were calculated using the Mann-Whitney U-tests, and *p < 0.05 was considered significant. Note that mRNA data is presented in dot-plot graphs with a log-scale, while the statistical analyses were performed on non-transformed data. PBMCs: Peripheral blood mononuclear cells

**Fig. 6:**
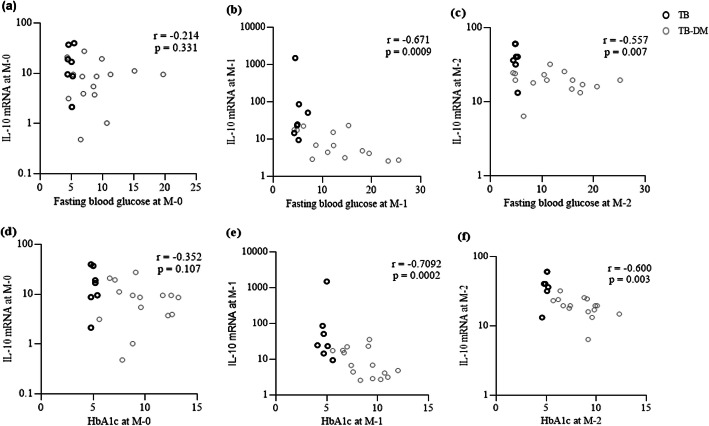
Correlation of sputum IL-10 transcript with fasting blood glucose and HbA1c levels. IL-10 transcript levels in sputum samples from TB and TB-DM patients combined (*n* = 22) were correlated with fasting blood glucose at (**a**) baseline and after (**b**) 1 month and (**c**) 2 months, and HbA1c levels at (**d**) baseline and after (**e**) 1 month and (**f**) 2 months of anti-TB treatment. Data is presented as median and the correlation was calculated using Spearman’s correlation test. *p < 0.05 was considered significant

CD4 and CD8 transcripts in PBMCs were not significantly different between the TB and TB-DM groups as assessed by Mann-Whitney U-test (Fig. 5a-b). In contrast, CD4 mRNA was significantly lower in sputum cells in TB-DM compared to TB patients both at baseline and after 1 month of anti-TB treatment (Fig. 5f), while the expression of CD8 transcripts were significantly higher in TB-DM patients at month-2 (Fig. 5g). Expression of the pro-inflammatory cytokines IL-1β and TNF-α in PBMCs decreased in the TB patients after 1 month of anti-TB treatment, with significantly lower expression seen in TB group compared to those in the TB-DM group at this time point (Fig. 5c-d). In the case of IL-1β, this difference remained after 2 months of anti-TB treatment (Fig. 5c). Contrary to the aforementioned results, there were no differences in IL-1β or TNF-α transcripts in the sputum between the groups at any time point (Fig. 5h-i). The tissue degrading enzyme MMP9 was higher in both PBMCs and sputum cells from TB-DM patients at baseline and at follow up compared to TB patients, although this trend was not significant (data not shown). Peripheral expression of the anti-inflammatory cytokine IL-10 was similar in TB-DM and TB patients at baseline, but TB-DM patients exhibited a significantly higher peripheral expression of IL-10 at month-2 compared to TB patients (Fig. 5e). In contrast, a significantly lower IL-10 expression in sputum cells was observed in TB-DM patients at months-1 and -2 (Fig. 5j). Furthermore, correlation analysis showed that high blood glucose and HbA1c levels were inversely correlated to IL-10 transcripts in sputum cells from both patient groups after 1 and 2 months of anti-TB treatment (Fig. 6a-f). These results suggest that TB-induced inflammation was not reduced as effectively in TB-DM after the start of anti-TB treatment when compared to TB patients, indicating that a low-grade inflammation persisted in TB-DM patients.

## Discussion

This longitudinal study explored the clinical, radiological and immune responses in patients with TB-DM compared to TB disease before and after start of anti-TB treatment. After onset of TB chemotherapy, TB-DM patients displayed a greater degree of lung pathology in the middle and lower lung zones compared to those with TB alone, despite similar clinical and microbiological responses to anti-TB treatment. A slower radiological improvement in TB-DM patients was accompanied with elevated transcript levels of the pro-inflammatory cytokines IL-1β and TNF-α as well as anti-inflammatory IL-10 in PBMCs, while in the lung, higher expression of CD8 and TNF-α transcripts were observed whereas CD4 and IL-10 were reduced. These results suggest that non-resolving systemic and local inflammation likely represent a persistent low-grade inflammation and an enhanced pulmonary involvement in TB-DM comorbidity that, in turn, may contribute to lung damage resulting from impaired counter regulatory mechanisms.

It was evident that TB-DM patients experienced poor glycemic control throughout the study period. Previous studies have found that TB infection can contribute to impaired glucose tolerance and worsened glycemic control in diabetic patients, and in some cases can even cause a new onset of diabetes [42, 43]. Elevated levels of fasting blood glucose and HbA1c in the present study could also result from poor compliance with dietary advice, diabetic drugs and/or exercise.

We did not observe any differences in sputum-smear positivity at enrollment between the groups, although a higher bacterial burden in sputum from TB-DM patients at diagnosis has frequently been observed [7, 10, 44]. Previously, it has been described that TB-DM co-morbidity could result in higher susceptibility to TB [1, 42], more severe clinical symptoms, and slower responses to anti-TB treatment including delayed sputum-culture conversion [6, 7, 44–46]. However, a few other studies reported no differences in sputum conversion rates or final treatment outcome between TB and TB-DM patients after anti-TB therapy [9, 10]. In the present study, sputum or culture conversion was not delayed in TB-DM patients compared to TB patients, and accordingly treatment outcome did not differ between the groups. Severity of clinical TB symptoms at baseline and during anti-TB treatment were similar in TB and TB-DM patients, and both groups demonstrated significant disease remission after the start of treatment, as reported in other studies [5, 47, 48]. Consistent with other reports, the socioeconomic status was more often higher in TB-DM patients [49], and comorbidity was associated with higher age and BMI compared to TB alone [5, 50, 51]. However, potential age-related immune dysfunctions or other physiological variables that are generally associated with older age groups (e.g. > 65 years) are less likely to occur in the present cohort, where the median age range is from 26.6 years to 40.1 years [52]. Although, our data analyses were adjusted for these confounders, the differences in baseline demographics may still have influenced the treatment outcome in this patient cohort. It was recently observed that TB patients in India with a low BMI had a considerably longer time to sputum culture conversion and enhanced rates of treatment failure and death when compared to patients with a normal-to-high BMI (independent of DM status) [53]. Thus, demographic variables such as BMI or socioeconomic status might obscure the negative effects of DM on TB.

It has been demonstrated that TB-DM patients exhibit atypical radiographic findings including a high frequency of lower lung involvement and more advanced forms of cavitary disease [54–56]. This study also observed an enhanced magnitude and duration of pathological lesions in the lung, especially in the middle and lower zones of the TB-DM patients (Fig. 4 and Table S3). However, neither TB-DM nor TB patients had cavitary TB, which is a deviation from the radiological findings in other studies examining TB-DM disease. Absence of lung cavities could result from less advanced TB disease in this cohort, which may also account for the lack of differences in the clinical and bacteriological response in TB-DM compared to TB patients. Some studies suggest that uncontrolled DM (HbA1c > 7%) is associated with TB disease progression including enhanced pathological lung involvement, despite no differences in clinical symptoms or sputum smear results between diabetic and non-diabetic TB patients [48, 54, 57]. We found a significant positive association between HbA1c levels and lung pathology when both TB and TB-DM patient groups were combined. Our observations suggest that the TB-DM patients enrolled in this study with either controlled or persistent hyperglycemia of ≤5 years duration and without other associated complications, had manifestations of a relatively mild TB disease. This in turn could reduce the possibility of detecting clinically relevant differences in TB symptoms and bacteriological outcomes in those with TB-DM compared with non-diabetic TB patients. In spite of this, the extent of lung pathology and local immune responses in the respiratory tract may be different in TB-DM compared to TB patients, which could represent early immune deviations in clinical TB disease in TB-DM patients. According to the guidelines of the Diabetic Association of Bangladesh (DAB), all DM patients attending BIRDEM or DAB linked centers/clinics are routinely asked about respiratory symptoms and consequently screened for pulmonary TB thereby leading to TB diagnosis in the early phase of disease. Thus, an early diagnosis and rapid implementation of adequate anti-TB treatment is likely important for the outcome of TB-DM disease.

According to the mRNA data (Fig. 5), peripheral expression of the proinflammatory cytokines TNF-α and IL-1β, as well as the anti-inflammatory mediator IL-10 was higher in TB-DM compared to TB patients after anti-TB treatment. These differences are primarily due to a down-regulation of these cytokine transcript levels in TB group but sustained expression in the TB-DM group. Excess IL-1β has been shown to promote neutrophil accumulation in the lung of active TB patients, which may exacerbate lung damage and disease progression [15]. Interestingly, a high-fat diet has been documented to induce an inflammatory IL-1β response that impairs insulin signaling and results in reduced glucose tolerance and insulin sensitivity, which is a hallmark of type 2 DM [58]. Accordingly, pharmacological blockade of IL-1β in patients with type 2 DM improved beta-cell secretory function and reduced hyperglycemia and markers of systemic inflammation [59]. Lower levels of CD4 and IL-10 mRNA in the lungs of TB-DM patients after treatment, but higher expression of CD8 and TNF-α are other important findings of the study, which may possibly explain the persistence seen in pulmonary inflammation during anti-TB therapy. It is likely that TB-DM patients may not be able to down-regulate pro-inflammatory responses as effectively because of insufficient local IL-10 production in the lungs. Enhanced IL-10 levels in the periphery but reduced IL-10 in sputum cells from TB-DM patients at follow up, may also indicate that IL-10 producing cells fail to migrate properly from the peripheral circulation to the Mtb-infected lung. In support of this, the low IL-10 production capacity of immune cells and reduced anti-inflammatory function of IL-10 have been reported in TB-DM disease compared to TB alone [60, 61]. While IL-10 could contribute to unwanted immunosuppression of Mtb-specific immune responses [62, 63], it is also necessary to control local pathological inflammation and sustain an environment that limits Mtb replication [64]. Moreover, it has also been shown that hypo-responsiveness to IL-10 signaling contributes to chronic low-grade inflammation in type 2 DM [65]. Similar to our findings, a recent study reported prolonged inflammation and delayed resolution of inflammation due to insufficient expression of anti-inflammatory cytokines including IL-10 in TB-DM compared to TB patients [13]. However, it was demonstrated in this study that hyperinflammation and low IL-10 levels were already present at baseline, with a slow resolution of inflammation in TB-DM patients [13]. In influenza virus infection, IL-10 producing CD4 cells have a critical function to limit pulmonary inflammation and tissue injury, and therefore, the blockade of T-cell derived IL-10 enhanced lethal inflammation without impacting virus titers in vivo [66]. Similarly, a decrease in IL-10 producing CD4 cells in the lungs of TB-DM patients may enhance inflammation without affecting the mycobacterial load in sputum. A recent longitudinal study using positron emission tomography and computerized tomography (PET-CT) to evaluate local lung inflammation in pulmonary TB patients demonstrated enhancement and/or development of new inflammatory lesions in many patients despite completion of 6 months of anti-TB therapy and 1 year of follow-up [67]. Patients that were reportedly culture negative at the end of treatment were found to have Mtb transcripts in sputum and bronchoalveolar lavage, suggesting that persistent bacterial transcription or stabilized mRNA from dormant/dead bacteria may fuel inflammatory reactions in the lung. It is possible that persistence of lung pathology in TB-DM comorbidity in the present study may result from either non-resolving sterile inflammation (high TNF-α and low IL-10 in the lungs) or persistence of non-replicating Mtb due to inefficient efferocytosis leading to inflammation [68, 69]. Further studies should confirm whether low IL-10 levels but enhanced proinflammatory cytokines could fuel lung pathology in TB-DM patients compared to TB patients and promote the progression towards severe cavitary disease.

The strengths of this study include longitudinal sampling of both peripheral blood and lung-derived sputum; the limitations were small patient numbers, age and sex imbalances as well as differences in BMI, SES and BCG vaccination status between the groups. According to the National TB prevalence survey 2015–2016, bacteriologically confirmed TB cases in Bangladesh are 2–3 time higher in males compared to females, which could explain why there was a higher enrollment of males in this study. Furthermore, the inclusion/exclusion criteria (e.g. the limitation of DM duration to 5 years) used in this study hampered complete matching of demographic parameters and therefore there was some bias in patient enrollment, which could also have affected the outcome. However, the requirement that only TB-DM patients with hyperglycemia of ≤5 years duration participated in the study allowed us to detect early inflammatory changes in TB-DM cases that may contribute to severe effects such as cavitary disease in the long term when left untreated. In addition, follow-up of TB-DM patients at 6 months instead of 9 months anti-TB therapy could have decreased the likelihood of detecting clinically significant differences between the groups. Another limitation in this study lies with the sputum sample collection method; expectorated sputum especially from the later time points (2 months), may mostly contain cells from the upper airways including both immune cells and oropharyngeal epithelial cells. Additionally, mRNA expression of phenotype markers and cytokines in the cells may not correlate directly with the protein expression of the same markers. Although to counteract this point, we have previously found that mRNA transcripts represent a good complementary measure to protein expression in tissue samples obtained from TB infected patients [37, 41].

## Conclusion

TB-DM disease was associated with a greater degree of pathological lung involvement in the lower lung areas after standard anti-TB treatment, despite similar clinical TB symptoms and sputum conversion rates. The results suggest that DM may reduce the control of persistent inflammation in TB through insufficient local IL-10 production in the lung, which could result in pulmonary impairment and aggravation of TB-DM disease.

## Supplementary information

**Additional file 1 Table S1.** Baseline clinical signs and symptoms of the study patients. **Table S2.** Hematological features in the TB and TB-DM patients and healthy controls. **Table S3**. Pulmonary pathology in TB-DM compared to TB patients.

## Data Availability

The datasets used in this study are available from the corresponding author on reasonable request.

## Abbreviations

AFB: Acid fast bacilli
BMI: Body mass index
CBC: Complete Blood Count
CPR-IR: C-peptide reactivity-insulin resistance
DM: Diabetes mellitus
DOTS: Directly observed therapy short-course
ESR: Erythrocyte sedimentation rate
GEE: Generalized estimating equation
HbA1c: Hemoglobin A1c
HOMA-IR: Homeostatic model assessment of insulin resistance
MLR: Monocyte-to-lymphocyte ratio
MMP: Matrix metalloproteinase
Mtb: *Mycobacterium tuberculosis*
NLR: Neutrophil-to-lymphocyte ratio
PBMC: Peripheral blood mononuclear cells
PLR: Platelet-to-lymphocyte ratio
SES: Socio-economic status
IL: Interleukin
TB: Tuberculosis
TNF: Tumor necrosis factor
WBC: White Blood Cells
