# Supplementary material for: Slow radiological improvement and persistent low-grade inflammation after chemotherapy in tuberculosis patients with type 2 diabetes
Source: BMC Infect Dis. 2020 Dec 7;20:933. doi: 10.1186/s12879-020-05473-x (PMC7722325; doi:10.1186/s12879-020-05473-x)
Supplement: Supplementary file 1 — Additional file 1 Table S1. Baseline clinical signs and symptoms of the study patients. Table S2. Hematological features in the TB and TB-DM patients and healthy controls. Table S3. Pulmonary pathology in TB-DM compared to TB patients. [file 12879_2020_5473_MOESM1_ESM.docx]

**Slow radiological improvement and persistent low-grade inflammation after chemotherapy in tuberculosis patients with type 2 diabetes**

Akhirunnesa Mily^1,2,a^, Protim Sarker^2,a^, Inin Taznin^2^, Md. Delwar Hossain^3^, Md. Ahsanul Haq^2^, SM. Mostofa Kamal^4^, Birgitta Agerberth^5^, Susanna Brighenti^1,b^ and Rubhana Raqib^2,b,^*

^1^Center for Infectious Medicine (CIM), Department of Medicine Huddinge, ANA Futura, Karolinska Institutet, Sweden

^2^Infectious Diseases Division, International Centre for Diarrhoeal Disease Research, Bangladesh, Dhaka, Bangladesh

^3^Respiratory Medicine, Bangladesh Institute of Research and Rehabilitation in Diabetes, Endocrine and Metabolic Disorders, Dhaka, Bangladesh

^4^National Institute of the Diseases of the Chest and Hospital, Dhaka, Bangladesh

^5^Clinical Microbiology, Department of Laboratory Medicine (Labmed), ANA Futura, Karolinska Institutet, Sweden

**E-mails:**

Akhirunnesa Mily: [mily.Akhirunnesa@ki.se](mailto:mily.Akhirunnesa@ki.se); Protim Sarker^:^ [protim@icddrb.org](mailto:protim@icddrb.org); Inin Taznin: [inin.taznin@yahoo.com](mailto:inin.taznin@yahoo.com); Delwar Hossain: [delwarhschest@yahoo.com](mailto:delwarhschest@yahoo.com); Md. Ahsanul Haq: [shohag@icddrb.org](mailto:shohag@icddrb.org), SM. Mostofa Kamal: [kamalsmmostofa@gmail.com](mailto:kamalsmmostofa@gmail.com); Birgitta Agerberth: [Birgitta.Agerberth@ki.se](mailto:Birgitta.Agerberth@ki.se); Susanna Brighenti: [Susanna.Brighenti@ki.se](mailto:Susanna.Brighenti@ki.se);

^a,b^ equal contribution

*Corresponding author:

Rubhana Raqib, PhD

Infectious Diseases Division,

International Centre for Diarrhoeal Disease Research, Bangladesh

Email: [rubhana@icddrb.org](mailto:rubhana@icddrb.org)

Phone: +880-2-9827068

**Supplementary Table 1.** Baseline clinical signs and symptoms of the study patients^a^

| Variables | TB (n=40) | TB-DM (n=40) | *p*-value |
| --- | --- | --- | --- |
| Cough | 40 (100) | 40 (100) | 0.991 |
| Hemoptysis | 18 (45.0) | 16 (40.0) | 0.30 |
| Dyspnoea | 0 | 4 (10.0) | 0.04 |
| Chest Pain | 36 (90.0) | 26 (65.0) | <0.001 |
| Night sweat | 27 (67.5) | 36 (90.0) | <0.001 |
| Anemia | 10 (33.3) | 0 | 0.001 |
| Fever | 40 (100) | 40 (100) | 0.991 |
| BMI (Kg/m2) |  |  |  |
| <18 | 26 (65.0) | 2 (5.0) | <0.001 |
| <16 | 9 (22.5) | 0 | 0.001 |
| MUAC |  |  |  |
| <220 mm | 9 (22.5) | 4 (10.0) | 0.390 |
| <200 mm | 2 (20.0) | 0 | 0.152 |
| Loss of appetite | 33 (82.5) | 22 (55) | 0.002 |
| Anorexia | 37 (92.5) | 33 (82.5) | 0.176 |
| Nausea | 29 (72.5) | 17 (42.5) | 0.004 |
| Joint pain | 16 (40.0) | 22 (55.0) | 0.179 |

^a^Data presented as number (percentage) of patients. Chi-square test was used to compare clinical variables between TB to TB-DM patients at baseline.

BMI: body mass index

**Supplementary Table 2.** Hematological features in the TB and TB-DM patients and healthy controls^a^

|  | Controls | TB group (n=35)^b^ | | | | TB-DM group (n=36)^b^ | | | |
| --- | --- | --- | --- | --- | --- | --- | --- | --- | --- |
|  | (n-20) | Baseline^c^ | Month 1^c^ | Month 2^c^ | Month 6^c^ | Baseline^c^ | Month 1^c^ | Month 2^c^ | Month 6^c^ |
| ESR | 22.56±5.9 | 38.95±4.3 | 38.64±4.0 | 29.21±3.7 | 11.30±2.2 | 44.0±4.31 | 26.70±3.7^§^ | 18.0±3.5 | 9.64±2.1 |
| Hb, mg/dL | 13.44±0.40 | 12.39±0.3 | 13.10±0.29 | 13.74±0.45 | 14.18±0.27 | 13.43±0.3* | 14.33±0.31^§^ | 14.18±0.42 | 15.00±0.26 |
| WBC count, % | 7.71±0.81 | 10.91±0.61 | 9.69±0.91 | 8.47±0.57 | 7.16±0.38^†^ | 11.86±0.60 | 9.80±0.59 | 8.76±0.57 | 8.66±0.39^†^ |
| Lymphocyte, % | 31.55±1.9 | 17.52±1.89 | 22.11±2.19 | 26.68±2.22 | 33.57±1.80 | 19.25±1.19 | 25.24±2.06 | 29.34±2.13 | 31.33±1.73 |
| Neutrophil, % | 58.30±2.57 | 73.01±1.73 | 65.27±2.49 | 63.11±2.29 | 55.39±1.90 | 71.25±1.73 | 64.87±2.34 | 59.71±2.20 | 58.60±1.82 |
| Monocyte, % | 5.46±0.47 | 6.51±0.33* | 5.23±0.26 | 5.64±0.33 | 5.78±0.26 | 5.36±0.34 | 5.21±0.25 | 5.01±0.32 | 4.58±0.20 |
| Platelet, % | 308.5±32.1 | 382.5±22.7 | 316.5±26.7 | 291.4±26.7 | 276.0±16.2 | 391.9±22.7 | 331.5±25.1 | 305.7±25.1 | 239.9±15.3 |
| NLR | 2.66±0.69 | 4.74±0.50 | 3.30±0.44 | 2.81±0.35 | 1.82±0.24 | 4.68±0.50 | 3.24±0.41 | 2.53±0.34 | 2.11±0.23 |
| MLR | 0.24±0.06 | 0.42±0.04 | 0.26±0.03 | 0.24±0.02 | 0.18±0.02 | 0.34±0.03 | 0.24±0.03 | 0.20±0.02 | 0.17±0.02 |
| PLR | 14.61±4.6 | 25.0±3.37 | 15.84±3.29 | 12.18±3.43 | 8.78±0.73 | 26.31±2.21 | 17.74±3.10 | 15.0±3.29 | 8.12±0.70 |

^a^Data was given with mean ± standard error of means (SEM).

^b^Multivariate regression analysis was used for comparisons between TB and TB-DM groups and the model was adjusted by age, sex, baseline BMI, SES score and BCG vaccination status. Between groups difference are depicted by the following symbols, at baseline*; month 1^§^; month 6^†^. Significant data at baseline were adjusted at the subsequent time points.

^c^Two-way repeated measure ANOVA was carried out to evaluate within group difference between baseline and month 1 or month 2 or month 6.

BCG: *Bacillus Calmette–Guérin;* BMI: body mass index; ESR: erythrocyte sedimentation rate; Hb: hemoglobin; MLR: monocyte-to-lymphocyte ratio; NLR: neutrophil-to-lymphocyte ratio; PLR: platelet-to-lymphocyte ratio; SES: socioeconomic status; WBC: white blood cell.

**Supplementary Table 3**. Pulmonary pathology in TB-DM compared to TB patients^a^

| % Lung  involvement^d^ | Month | Difference (95% CI) | p-value |
| --- | --- | --- | --- |
| Whole lung | 0 | 8.03 (0.01, 16.05) | 0.050^b^ |
|  | 1 | 0.55(-4.01, 5.12) | 0.811^b^ |
|  | 2 | 1.18(-3.86, 6.22) | 0.641^b^ |
|  | 6 | -1.00(-4.72, 2.72) | 0.584^b^ |
|  | Overall changes | 6.34(0.33, 12.35) | 0.039^c^ |
| Upper zone | 0 | -11.20(-37.00, 14.55) | 0.389^b^ |
|  | 1 | 6.13(-18.77, 31.02) | 0.625^b^ |
|  | 2 | 3.86(-17.37, 25.09) | 0.718^b^ |
|  | 6 | 0.87(-15.48, 17.22) | 0.916^b^ |
|  | Overall changes | -6.30(-24.90, 12.30) | 0.507^c^ |
| Middle zone | 0 | 29.36(6.53, 52.18) | 0.012^b^ |
|  | 1 | 5.41(-1.73, 12.56) | 0.135^b^ |
|  | 2 | 4.80(-5.83, 15.43) | 0.371^b^ |
|  | 6 | -1.41(-13.04, 10.22) | 0.810^b^ |
|  | Overall changes | 25.37(7.92, 42.82) | 0.004^c^ |
| Lower zone | 0 | 23.76(4.11, 43.40) | 0.018^b^ |
|  | 1 | -2.32(-9.46, 4.81) | 0.518^b^ |
|  | 2 | -1.12(-10.00, 7.74) | 0.801^b^ |
|  | 6 | 0.42(-8.22, 9.06) | 0.923^b^ |
|  | Overall changes | 16.83(2.65, 31.01) | 0.020^c^ |

^a^The results are expressed as difference of mean and 95% confidence interval (CI). ^b^Multivariate regression and ^c^Generalized estimating equation (GEE; for overall changes) models were used to compare TB (n=35) vs TB-DM (n=36) group and Both models were adjusted by age, sex, baseline BMI, SES score and BCG vaccination status and baseline lung involvement. GEE model was additionally adjusted by time (to reduce multicollinearity).

^d^Each of the three zones in the two lungs could have a maximum of 100% pathological involvement, and therefore the total % lung involvement in the upper, middle or lower zones respectively could be maximum 100 + 100 = 200%, while the total % lung involvement including all three zones in both lung lobes in a patient could be maximum 3 x 100 x 2 = 600%.
